# Ecological immunogenetics of life-history traits in a model amphibian

**DOI:** 10.1098/rsbl.2011.0845

**Published:** 2011-11-23

**Authors:** Seth M. Barribeau, Jandouwe Villinger, Bruce Waldman

**Affiliations:** 1 Experimental Ecology, Institute of Integrative Biology, ETH Zürich, Zürich 8092, Switzerland; 2 Molecular Biology and Bioinformatics Unit, International Centre of Insect Physiology and Ecology, PO Box 30772-00100, Nairobi, Kenya; 3 Department of Ecology, PO Box 84, Lincoln University, Canterbury 7647, New Zealand; 4 Laboratory of Behavioral and Population Ecology, School of Biological Sciences, Seoul National University, 1 Gwanak-ro, Gwanak-gu, Seoul 151-747, South Korea

**Keywords:** host–pathogen interactions, kin association, life history, local adaptation, major histocompatibility complex

## Abstract

Major histocompatibility complex (MHC) genes determine immune repertoires and social preferences of vertebrates. Immunological regulation of microbial assemblages associated with individuals influences their sociality, and should also affect their life-history traits. We exposed *Xenopus laevis* tadpoles to water conditioned by adult conspecifics. Then, we analysed tadpole growth, development and survivorship as a function of MHC class I and class II peptide-binding region amino acid sequence similarities between tadpoles and frogs that conditioned the water to which they were exposed. Tadpoles approached metamorphosis earlier and suffered greater mortality when exposed to immunogenetically dissimilar frogs. The results suggest that developmental regulatory cues, microbial assemblages or both are specific to MHC genotypes. Tadpoles may associate with conspecifics with which they share microbiota to which their genotypes are well adapted.

## 1. Introduction

Pathogens and hosts are locked in antagonistic coevolution as increased fitness for one results in reduced fitness for the other. Although host adaptations lag behind pathogens in this race owing to longer host generation times, hosts may be less susceptible to local pathogen repertoires to which they have had an opportunity to evolve defences [1]. Local microbial assemblages correlate with differences in hosts' adaptive immune genes [2], but how these ‘personal microbiota’ affect fitness is unknown. Locally adapted pathogens are more readily transmitted among and can be more virulent to genetically similar hosts [3]. In contrast, virulence of locally adapted microbiota may increase in new hosts [4].

The vertebrate major histocompatibility complex (MHC) encodes cellular recognition of pathogens and influences mating and social behaviour. MHC genotypes of individuals determine immunorecognition of different pathogen repertoires [5]. Host-specific immunological responses shape individuals' microbiota, both commensal and parasitic [6,7]. These, in turn, produce cues used for social recognition [8]. Mating preferences for MHC-dissimilar partners should facilitate inbreeding avoidance and make offspring less vulnerable to disease by increasing their heterozygosity or by shuffling MHC alleles to confer protection against pathogens that successfully exploit parental genotypes [5]. Social preferences for MHC similarity facilitate cooperation among kin. Examples include communal nesting partner preferences in mice [9] and schooling preferences in tadpoles [10]. Thus, social behaviour both influences and is influenced by the micro-organisms with which individuals live [11].

When associating with MHC-similar conspecifics, individuals may benefit from exposure to low-virulence micro-organisms that are adapted to their immune system [6]. However, new pathogens may pose a significant risk by exploiting shared weaknesses in group members' immune systems and thus spread rapidly. In contrast, associating with MHC-dissimilar conspecifics may expose individuals to novel highly virulent micro-organisms. Whether MHC-biased social behaviours are adaptive cannot be fully understood without first determining their immunological costs and benefits, both affecting the individual itself and the conspecifics with which it interacts [12].

To determine the fitness consequences of MHC-based association, we exposed *Xenopus laevis* tadpoles to water conditioned by adult conspecifics. Tadpoles and adults of this species share the same habitat and microbial environment. MHC loci regulate both immunity to bacteria [13] and association preferences among tadpoles [10]. We assessed three life-history traits—growth, development and survivorship—as a function of the MHC similarity between adults and tadpoles.

## 2. Material and methods

We bred *X. laevis* with known MHC class I and class II sequences (haplotypes *f, g* and *j* [14,15]; GenBank accession numbers: class I AF185580, AF185579 and AF185586; class II AF454374, AF454377 and AF454376). The frogs originated from the Basel Institute for Immunology and had been bred for several generations in our laboratory to maintain shared background genetic variation in frogs homozygous for different MHC haplotypes. All frogs were maintained in 60 l holding tanks into which water was fed continuously (3 l h^−1^). The flow-through system was fed by filtered, aerated water (21°C) sourced from a deep aquifer.

We mated six adult MHC-homozygous frogs (*ff*, *gg*, *jj*) twice in one night to obtain tadpoles of six MHC genotypes (*ff*, *fg*, *fj*, *gg*, *gj*, *jj*) [13]. We induced oviposition by injecting the females into the dorsal lymph sac with 100 IU of luteinizing hormone-releasing hormone (Argent Chemical Laboratories, Redmond, WA, USA). MHC-identical frogs were paired first, after which amplexed adults were separated and paired with MHC-dissimilar partners. After eggs hatched, we placed 100 tadpoles from each brood into 10 l tanks.

Subsequently, we isolated the maternal frogs, and two additional MHC heterozygous females (*fg*, *gj*), in 60 l flow-through tanks and fed them sliced ox liver every 2 days. After 4 days, we stopped the water flow to allow chemical cues and micro-organisms to accumulate, and ceased feeding the frogs to limit fouling. To obtain an estimate of microbial density, we plated 10 µl of the water from each tank in triplicate onto tryptone soya agar (Oxoid, Basingstoke, UK), incubated the plates aerobically at 32°C, and counted the number of bacterial colonies at 24 and 48 h. We exposed tadpoles to water from frogs, standardized by dilution to the microbial concentration of the lowest number of colonies found (*gg* female: 250 cfu ml^−1^).

Two weeks after hatching, we separated 300 tadpoles, 50 of each genotype, individually into beakers containing 1 l of conditioned water. Ten tadpoles of every genotype (*ff*, *fg*, *fj*, *gg*, *gj*, *jj*) were exposed to water from each of *ff*, *fg*, *gg*, *gj* and *jj* adults. We randomly assigned the tadpoles into six blocks. We fed tadpoles every 2 days with ground nettle suspension, topped the water up to 1 l every 4 days to replenish evaporation, and moved each beaker one place each day to limit position effects. Three weeks after exposure, we recorded developmental stage [16] and photographed each tadpole from 60 cm above to measure snout–vent length (SVL) using ImageJ v. 1.3 (NIH, Bethesda, MD, USA).

We analysed how tadpole mortality varied with the number of shared MHC haplotypes (0/1/2; *n* = 110/140/50) and block by binomial generalized linear models (GLM) using R v. 2.10 (www.r-project.org). We examined how tadpole development varied in response to the percentage of shared amino acids at MHC class I and II loci peptide-binding region (PBR) domains [14,15] by quadratic regressions with tadpole SVL as a covariate. Developmental stage was analysed with Gaussian GLMs including SVL as a covariate. We also analysed the effects of relatedness of frogs (whether water was conditioned by the maternal parent), and the ratio by which conditioned water was diluted, on tadpole size, developmental stage and mortality in additional GLMs.

## 3. Results

**Figure 1.**
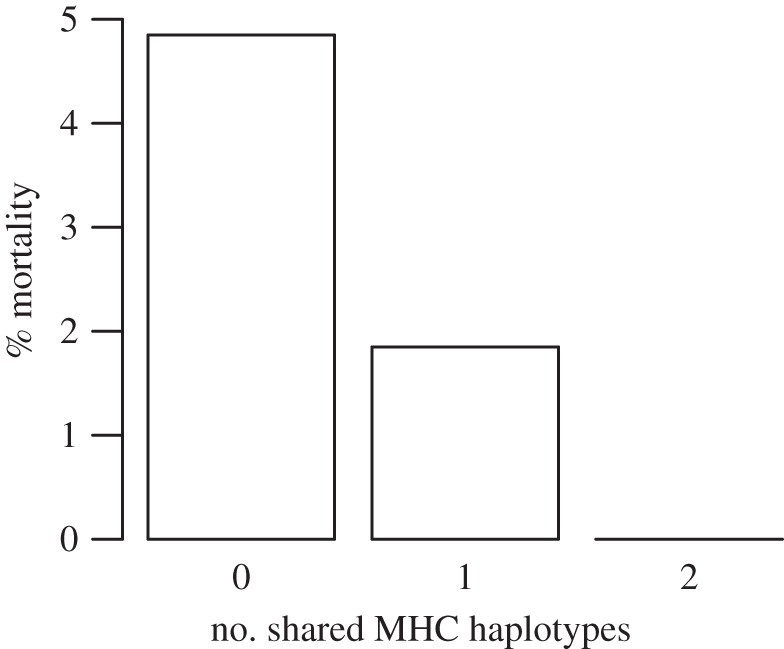
Tadpole mortality decreased with the number of MHC haplotypes that tadpoles shared with frogs that conditioned exposure water.

Tadpole mortality decreased with the number of MHC haplotypes shared with frogs that had conditioned the water (figure 1; 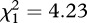, *p* = 0.04). Three times as many tadpoles died when exposed to water conditioned by frogs with which they shared no haplotypes compared with when they shared one haplotype. No tadpoles died when reared in water conditioned by MHC-identical donor frogs (figure 1).

**Figure 2.**
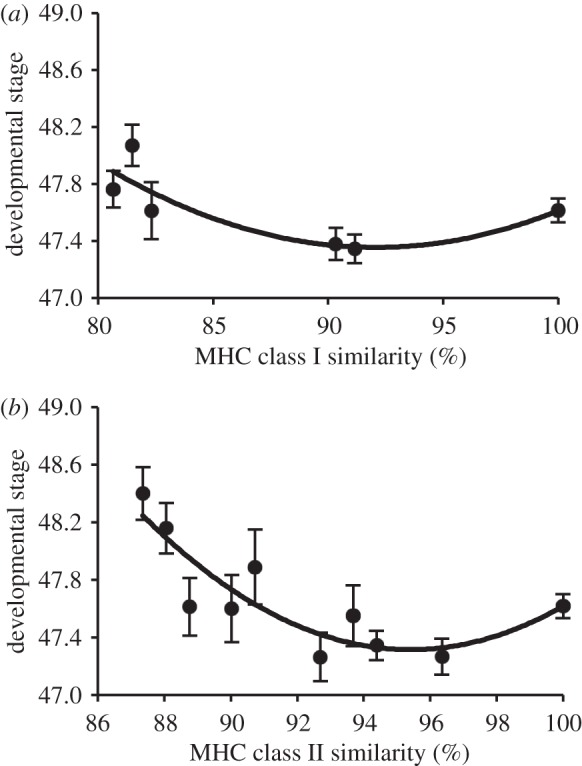
Developmental stage (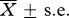) [16] decreased as a function of MHC PBR sequence similarity between tadpoles and frogs that conditioned water, in both (*a*) class I and (*b*) class II loci. Lines represent quadratic polynomial regressions. Greater developmental stage indicates accelerated development and metamorphosis.

Tadpole development slowed in proportion to the number of shared MHC haplotypes (*F*_1,291_ *=* 4.31, *p* = 0.039) and PBR amino acid sequence similarity (figure 2*a*, class I: *t*_289_ = 3.09, *p* = 0.0022, overall *F*_3,289_ = 4.15, *p* = 0.0067; class II: *t*_289_ = 4.92, *p* < 0.0001, overall *F*_3,289_ = 10.80, *p* < 0.0001). With increasing PBR sequence similarity, development at first slowed to a minimum (95% similarity, MHC class II) and then increased (figure 2*a*,*b*).

Tadpole size varied independently of developmental stage (*F*_1,290_ = 0.07, *p* = 0.80) and the number of shared MHC haplotypes (*F*_1,291_ = 0.62, *p* = 0.29). Nor did tadpole size explain stage differences in our regression analyses (class I: *t*_289_ = 0.33, *p* = 0.74; class II: *t*_289_ = 0.46, *p* = 0.65). Kinship cues and dilution factor did not significantly influence development (relatedness: *F*_1,291_ = 0.85, *p* = 0.36; dilution: *F*_1,291_ = 0.11, *p* = 0.74), growth (relatedness: *F*_1,291_ = 0.18, *p* = 0.67; dilution: *F*_1,291_ = 1.75, *p* = 0.19) or survival (relatedness: 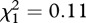, *p* = 0.74; dilution: 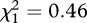, *p* = 0.50).

## 4. Discussion

More tadpoles died in water conditioned by MHC-dissimilar than MHC-similar frogs, but surviving tadpoles developed faster. Tadpoles exposed to cues from conspecifics dissimilar to themselves in MHC type suffered reduced fitness. These results demonstrate that the MHC identity of conspecifics with which individuals interact not only influences the social behaviour but also life-history traits of group members.

Our study does not establish the mechanism by which the MHC affects development or survivorship. Possibly, developmental regulatory cues, such as those thought to modulate tadpole growth [17], specifically target and repress development of immunogenetically dissimilar conspecifics. Alternatively, individuals and the microbial assemblages associated with them may become co-adapted over time. Such personal microbiota, when transferred to new immunologically dissimilar hosts, might increase in virulence. These hypotheses are not mutually exclusive.

Tadpoles exposed to cues of immunogenetically dissimilar frogs developed faster without growing correspondingly larger. Such accelerated development causing tadpoles to approach metamorphosis at smaller sizes typically occurs in unfavourable conditions. In *Xenopus*, more rapid metamorphosis also can be induced by exposure to predators, resulting in smaller juveniles [18]. In other anurans, such individuals suffer a reduced likelihood of survival and reproduction [19]. Adults may be more resistant to infection than tadpoles, which might favour rapid metamorphosis in some conditions. However, immunity is suppressed during metamorphosis, hence vulnerability to disease is then high [20].

Selection for a social environment with a compatible microbiota may explain the preferential association of tadpoles with others sharing the same MHC alleles [10]. MHC-similar individuals are more likely to transmit locally adapted pathogens that express low virulence. Individuals thereby might benefit from avoiding novel pathogens associated with immunologically dissimilar conspecifics. The advantages of preferential association with immunogenetically similar conspecifics might extend beyond pathogens to confer protection against other types of parasites as well [21]. However, because costs and benefits are environmentally modulated, we do not expect MHC-biased social assortment to be invariably selected nor expressed.

In sub-Saharan Africa, adult and larval *X. laevis* often live in turbid water. Little is known about their social behaviour in the wild [22], although adults cannibalize tadpoles in the laboratory. Thus, we cannot be certain of the functional significance of our results. Moreover, we determined neither the microbial composition nor the proportion of resident and transient microbiota [7] of water conditioned by adults. Resident microbiota would be more likely to be adapted to host frogs' immune systems than transient, possibly pathogenic, organisms.

Social groups are dynamic; individuals are exposed to a changing array of micro-organisms that vary with group composition and shifting environmental factors. Our results suggest that immunogenetic identity is important in assessing the costs and benefits of sociality. How microbiota associated with specific MHC genotypes, spanning the full spectrum from mutualistic to pathogenic organisms, affect the fitness of hosts and their conspecifics remains fertile ground for future work.

## References

[1] Ebert D. Hamilton W. D. 1996 Sex against virulence: the coevolution of parasitic diseases. Trends Ecol. Evol. 11, 79–82 10.1016/0169-5347(96)81047-0 (doi:10.1016/0169-5347(96)81047-0) 21237766

[2] Dionne M. Miller K. M. Dodson J. J. Caron F. Bernatchez L. 2007 Clinal variation in MHC diversity with temperature: evidence for the role of host–pathogen interaction on local adaptation in Atlantic salmon. Evolution 61, 2154–2164 10.1111/j.1558-5646.2007.00178.x (doi:10.1111/j.1558-5646.2007.00178.x) 17767587

[3] Ebert D. 1994 Virulence and local adaptation of a horizontally transmitted parasite. Science 265, 1084–1086 10.1126/science.265.5175.1084 (doi:10.1126/science.265.5175.1084) 17832903

[4] Qu A. 2008 Comparative metagenomics reveals host specific metavirulomes and horizontal gene transfer elements in the chicken cecum microbiome. PLoS ONE 3, e2945 10.1371/journal.pone.0002945 (doi:10.1371/journal.pone.0002945) 18698407 PMC2492807

[5] Apanius V. Penn D. Slev P. R. Ruff L. R. Potts W. K. 1997 The nature of selection on the major histocompatibility complex. Crit. Rev. Immunol. 17, 179–224 9094452 10.1615/critrevimmunol.v17.i2.40

[6] Feng T. Elson C. O. 2011 Adaptive immunity in the host–microbiota dialog. Mucosal. Immunol. 4, 15–21 10.1038/mi.2010.60 (doi:10.1038/mi.2010.60) 20944557 PMC4557730

[7] McKenzie V. J. Bowers R. M. Fierer N. Knight R. Lauber C. L. In press. Co-habiting amphibian species harbor unique skin bacterial communities in wild populations. ISME J. (doi:10.1038/ismej.2011.129) 10.1038/ismej.2011.129 PMC3280140 21955991

[8] Lanyon C. V. Rushton S. P. O'Donnell A. G. Goodfellow M. Ward A. C. Petrie M. Jensen S. P. Gosling L. M. Penn D. J. 2007 Murine scent mark microbial communities are genetically determined. FEMS Microbiol. Ecol. 59, 576–583 10.1111/J.1574-6941.2006.00252.X (doi:10.1111/J.1574-6941.2006.00252.X) 17381516

[9] Manning C. J. Wakeland E. K. Potts W. K. 1992 Communal nesting patterns in mice implicate MHC genes in kin recognition. Nature 360, 581–583 10.1038/360581a0 (doi:10.1038/360581a0) 1461279

[10] Villinger J. Waldman B. 2008 Self-referent MHC type matching in frog tadpoles. Proc. R. Soc. B 275, 1225–1230 10.1098/rspb.2008.0022 (doi:10.1098/rspb.2008.0022) PMC2602699 18285278

[11] Archie E. A. Theis K. R. 2011 Animal behaviour meets microbial ecology. Anim. Behav. 82, 425–436 10.1016/j.anbehav.2011.05.029 (doi:10.1016/j.anbehav.2011.05.029)

[12] Cotter S. C. Kilner R. M. 2010 Personal immunity versus social immunity. Behav. Ecol. 21, 663–668 10.1093/Beheco/Arq070 (doi:10.1093/Beheco/Arq070)

[13] Barribeau S. M. Villinger J. Waldman B. 2008 Major histocompatibility complex based resistance to a common bacterial pathogen of amphibians. PLoS ONE 3, e2692 10.1371/journal.pone.0002692 (doi:10.1371/journal.pone.0002692) 18629002 PMC2443284

[14] Liu Y. Kasahara M. Rumfelt L. L. Flajnik M. F. 2002 *Xenopus* class II *A* genes: studies of genetics, polymorphism, and expression. Dev. Comp. Immunol. 26, 735–750 10.1016/S0145-305X(02)00034-4 (doi:10.1016/S0145-305X(02)00034-4) 12206837

[15] Flajnik M. F. Ohta Y. Greenberg A. S. Salter-Cid L. Carrizosa A. Du Pasquier L. Kasahara M. 1999 Two ancient allelic lineages at the single classical class I locus in the *Xenopus* MHC. J. Immunol. 163, 3826–3833 10490981

[16] Nieuwkoop P. D. Faber J. 1956 Normal table of *Xenopus laevis* (Daudin). Amsterdam, The Netherlands: North-Holland

[17] Waldman B. 1986 Chemical ecology of kin recognition in anuran amphibians. In Chemical signals in vertebrates 4: ecology, evolution and comparative biology (eds Duvall D. Müller-Schwarze D. Silverstein R. M.), pp. 225–242 New York, NY: Plenum Press

[18] Walsh P. T. Downie J. R. Monaghan P. 2008 Predation-induced plasticity in metamorphic duration in *Xenopus laevis*. Funct. Ecol. 22, 699–705 10.1111/j.1365-2435.2008.01429.x (doi:10.1111/j.1365-2435.2008.01429.x)

[19] Alford R. A. 1999 Ecology: resource use, competition, and predation. In Tadpoles: the biology of anuran larvae (eds McDiarmid R. W. Altig R.), pp. 240–278 Chicago, IL: University of Chicago Press

[20] Rollins-Smith L. A. 1998 Metamorphosis and the amphibian immune system. Immunol. Rev. 166, 221–230 10.1111/j.1600-065X.1998.tb01265.x (doi:10.1111/j.1600-065X.1998.tb01265.x) 9914915

[21] Jackson J. A. Tinsley R. C. 2005 Geographic and within-population structure of variable resistance to parasite species and strains in a vertebrate host. Int. J. Parasitol. 35, 29–37 10.1016/j.ijpara.2004.10.017 (doi:10.1016/j.ijpara.2004.10.017) 15619513

[22] Tinsley R. C. Kobel H. R. (eds) 1996 The biology of *Xenopus*. Oxford, UK Clarendon Press

